# Microbiota-Derived Metabolites, Indole-3-aldehyde and Indole-3-acetic Acid, Differentially Modulate Innate Cytokines and Stromal Remodeling Processes Associated with Autoimmune Arthritis

**DOI:** 10.3390/ijms22042017

**Published:** 2021-02-18

**Authors:** David Langan, Darren J. Perkins, Stefanie N. Vogel, Kamal D. Moudgil

**Affiliations:** 1 Department of Microbiology and Immunology, University of Maryland School of Medicine, Baltimore, MD 21201, USA; David.Langan@som.umaryland.edu (D.L.); dperkins@som.umaryland.edu (D.J.P.); Svogel@som.umaryland.edu (S.N.V.); 2 Research Service, Baltimore Veterans Affairs Medical Center, Baltimore, MD 21201, USA; 3 Department of Medicine, Division of Rheumatology and Clinical Immunology, University of Maryland School of Medicine, Baltimore, MD 21201, USA

**Keywords:** rheumatoid arthritis, microbiota, indole-3-acetic acid, indole-3-aldehyde, aryl hydrocarbon receptor, NF-κB, MAPK, innate inflammation, cytokines, angiogenesis, osteoclastogenesis

## Abstract

Rheumatoid arthritis (RA) is an autoimmune disease characterized by chronic inflammation of the synovial joints. Inflammation, new blood vessel formation (angiogenesis) and bone resorption (osteoclastogenesis) are three key processes involved in the joint damage and deformities of arthritis. Various gut microbiota-derived metabolites are implicated in RA pathogenesis. However, there is barely any information about the impact of two such metabolites, indole-3-aldehyde (IAld) and indole-3-acetic acid (I3AA), on arthritis-related processes. We conducted a comparative analysis of IAld and I3AA using established cell-based models to understand how they might influence RA pathogenesis. Although structurally similar, the bioactivities of these two metabolites were profoundly different. IAld but not I3AA, inhibited the expression of pro-inflammatory cytokines (IL-1β and IL-6) in RAW 264.7 (RAW) cells stimulated with heat-killed *M. tuberculosis* sonicate (Mtb) and lipopolysaccharide (LPS). IAld also exhibited pro-angiogenic activity and pro-osteoclastogenic activity. In contrast, I3AA exhibited anti-angiogenic activity on endothelial cell tube formation but had no effect on osteoclastogenesis. Both IAld and I3AA have been proposed as aryl hydrocarbon receptor (AhR) agonists. Use of CH-223191, an inhibitor of the AhR, suppressed the anti-angiogenic activity of I3AA but failed to mitigate the effects of IAld. Further investigation of the anti-inflammatory activities of IAld and I3AA in LPS-treated RAW cells indicated that inhibition of MyD88-dependent activation of NF-κB and MAPK pathways was not likely involved. Our results suggest that the relative bioavailability of these indole derivatives may differentially impact RA progression and possibly other diseases that share similar cellular processes.

## 1. Introduction

Rheumatoid arthritis (RA) is characterized by chronic inflammation of the synovial lining of the joints (synovitis) and damage to the cartilage and bone therein [1,2]. Inflammation, bone resorption (osteoclastogenesis) and new blood vessel formation (angiogenesis) represent three of the key cellular processes involved in arthritis pathogenesis [1,2,3,4]. Similar processes are implicated in certain other autoimmune diseases and cancer, although to varying degrees of involvement. In RA, macrophages (Mɸ) upregulate Toll-like Receptors (TLR) TLR2 and TLR4 [5]. TLR ligands in the synovium (e.g., high mobility group box 1 (HMGB1), fibrinogen and peptidoglycans) stimulate Mɸ and other synovial infiltrating cells [6,7,8], in part through Myeloid Differentiation Primary Response 88 (MyD88)-dependent signaling and downstream activation of nuclear factor-kappa B (NF-κB) and mitogen-activated protein kinase (MAPK) transcriptional pathways. As a result, IL-1β, IL-6 and TNFα, as well as other pro-inflammatory cytokines [9], are upregulated. These cytokines are central regulators of inflammation and subsequent joint destruction. As such, they have been successfully targeted for therapeutic purposes in RA [10].

Bone remodeling involves a fine balance between bone formation by osteoblasts and bone resorption by osteoclasts. In health, for example, bone resorption may increase to keep pace with bone formation for proper bone sculpting [11]. However, in disease conditions, increased osteoclastogenesis and increased expression of bone resorbing enzymes, namely cathepsin K (CatK) and tartrate-resistant acid phosphatase (TRAP), by these cells contribute to the formation of resorptive pits where osteoclasts associate with the bone. Osteoclasts are derived from myeloid precursors and, in a disease state, directly from Mɸ, when pro-inflammatory cytokines as well as receptor activator of NF-κB-ligand (RANKL) and macrophage-colony stimulatory factor (M-CSF) levels are elevated [12]. Angiogenesis is required to provide nutrition and growth factors to the growing bones in health. However, in arthritic joints, angiogenesis supports bone resorption by enhancing the recruitment of pathological innate immune cells, including Mɸ, as well as supporting the expansion of osteoclasts. Osteoclasts-derived matrix metalloproteinase 9 (MMP9) has also been described to enhance angiogenesis [13]. Interestingly, as with the innate inflammatory response, these stromal remodeling processes are also regulated by NF-κB and MAPK pathways. Thus, the cellular and molecular mediators of the above processes are potential therapeutic targets in autoimmunity and cancer. 

Microbiome ‘dysbiosis’ is linked to autoimmunity and cancer. In RA, ‘dysbiosis’ of the gut microbiota is thought to be partially corrected after certain clinical treatments [14,15]. Microbial-derived bioactive metabolites are largely responsible for mediating the microbiome’s effect on health and disease [16,17]. In part, the bioavailability of these metabolites is regulated by the diet and the diet is known to modulate the intestinal microbiota in RA patients and the elderly [18,19]. Short-chain fatty acids (SCFAs) are among the most well-characterized microbiota-derived metabolites that have been analyzed for their role in health and disease [20], whereas indole derivatives produced from tryptophan (Trp) have been largely understudied. In a recent study, two such indole derivatives, indole-3-acetic acid (I3AA) and indole-3-acetylaldehyde, were found to be decreased in the synovial fluid of RA compared to osteoarthritis (OA) patients [21]. In addition, restoration of bacterial strains that produce indole derivatives ameliorated adjuvant-induced arthritis, a rat model of RA [22]. Furthermore, the potential benefits afforded by indole derivatives are highlighted by the amelioration of diseases in animal models of colitis and experimental autoimmune encephalomyelitis (EAE) [23,24]. However, it is not yet clear whether indole derivatives can modulate the cellular processes involved in arthritis pathogenesis.

Several of the indole derivatives produced by the gut microbiota, including I3AA and indole-3-aldehyde (IAld) (Structures are shown in Figure S1), are suggested ligands for the aryl hydrocarbon receptor (AhR), a xenobiotic response receptor [25]. Notably, endogenous Trp metabolism and several resulting metabolites are AhR ligands that have been implicated in autoimmunity and cancer [21,26,27,28]. However, recent direct analyses of I3AA and IAld suggests that they are relatively weak agonists of the AhR compared to classical AhR agonists like 2,3,7,8-Tetrachlorodibenzo-p-dioxin (TCDD) [29,30,31]. As such, certain bioactivities of IAld and I3AA may be dependent on other pathways, for example NF-κB and MAPKs. Thus, previous observations on the immunomodulatory activities of indole derivatives warrant further investigation of how I3AA and IAld may influence the disease-related processes in autoimmune arthritis.

Here, we have used classical cell-based models of innate inflammation, osteoclastogenesis and angiogenesis to compare the effects of two microbial-indole derivatives, IAld and I3AA, on these RA-associated cellular processes. To test the effects of these metabolites on the innate inflammatory response of the murine RAW 264.7 (RAW) Mɸ cell line, we used heat-killed *M. tuberculosis* (Mtb), a TLR2 and TLR4 dual-agonist [32], which also is used as an arthritogen in certain animal models of RA [33] and lipopolysaccharide (LPS), a TLR4 agonist, which is a prototypic inducer of innate inflammation. We tested whether regulation of the AhR and MyD88-dependent pathways were involved in the tested bioactivities of IAld and I3AA. Furthermore, we examined stromal remodeling by employing assays involving RANKL-induced differentiation of RAW cells into osteoclasts and endothelial cell 2D-tube formation. Our findings highlight the differential bioactivities of IAld and I3AA and point to the potential relevance they may have to autoimmune arthritis and other diseases that share similar cellular processes.

## 2. Results

### 2.1. IAld But Not I3AA, Inhibits Expression of Several Inflammatory Cytokines by Mɸ in Response to Mtb 

**Figure 1:**
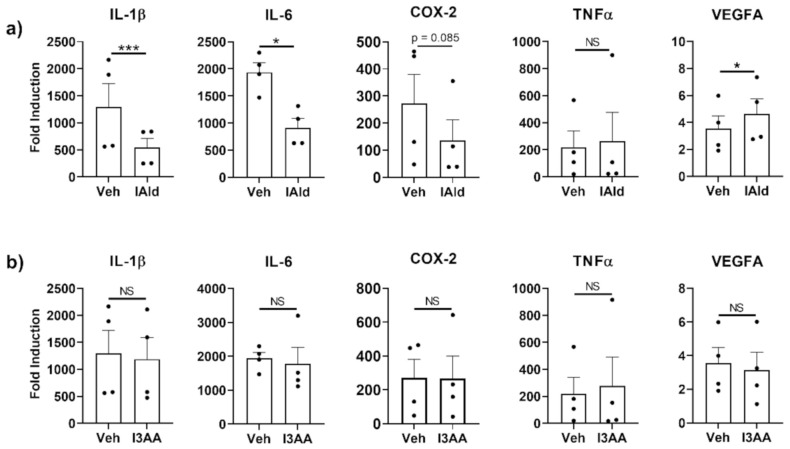
Indole-3-aldehyde (IAld) inhibits the production of subsets of pro-inflammatory cytokines and other mediators by RAW cells in response to *M. tuberculosis* (Mtb). RAW cells were treated with vehicle (Veh) or IAld (0.25 mM) (**a**) or with Veh or I3AA (0.25 mM) (**b**), 1 h before the addition of Mtb sonicate (10 μg/mL), followed by a 16 h incubation. Gene expression relative to the untreated vehicle control (Fold Induction) is reported. Data reported are from the combined results (Mean ± SEM) of 4 independent experiments (*n* = 4) whose values are shown as individual dots. Statistical difference between two groups in each panel was determined by a paired Student’s *t* test. Two- tailed *p*-value: NS = Not significant, * *p* ≤ 0.05 and *** *p* ≤ 0.001.

The expression of genes encoding various mediators of inflammation was measured by qRT-PCR in RAW cells (murine Mɸ) stimulated with Mtb sonicate, which induces TLR2-and TLR4-specific responses, in the absence or presence of IAld or I3AA. Steady-state mRNA levels of several inflammation-associated cytokines were upregulated by Mtb (Figure 1) but the induction of a subset of these were reduced by concomitant treatment with IAld (0.25 mM). Inhibited cytokines include IL-1β (*p* = 0.0013) and IL-6 (*p* = 0.0195) (Figure 1a). Additionally, there was a trend towards reduction of COX-2 mRNA but the change was not statistically significant (*p* = 0.085). In contrast, TNF-α was unaffected, whereas transcription of vascular endothelial growth factor A (VEGFA), a critical inducer of angiogenesis, was increased (*p* = 0.0183) in the presence of IAld. In contrast to the effects of IAld, concomitant I3AA treatment failed to alter the levels of any of the above-mentioned mediators of inflammation compared to Mtb-treated controls (Figure 1b). Use of I3AA at higher concentrations (up to 1.0 mM) showed similar results. Importantly, neither IAld nor I3AA exhibited any cytotoxic effect on RAW cells at the concentration (0.25 mM) used in these experiments (Figure S2). IAld showed additional anti-inflammatory potential by suppressing IL-6 expression by NIH3T3 fibroblast in response to IL-1β (*p* = 0.020); whereas, I3AA did not affect the expression of the measured cytokine response (Figure S3). 

### 2.2. IAld-Induced Inhibition in Response to Mtb was Partially Reversed by AhR Antagonist CH-223191

**Figure 2:**
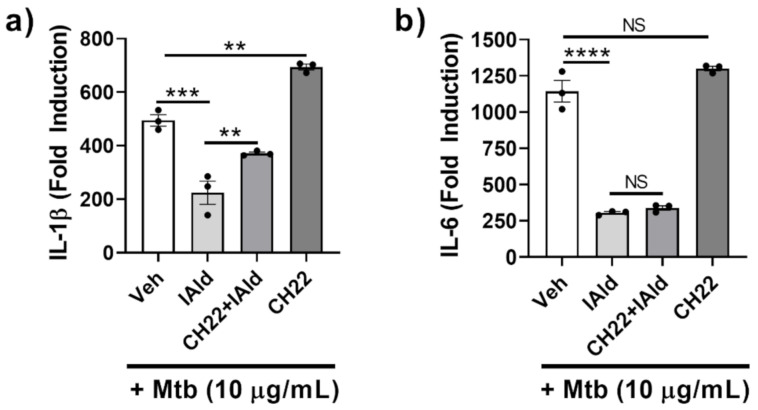
Aryl hydrocarbon receptor (AhR) antagonist CH-223191 partially reversed IAld-induced inhibition of IL-1β but not IL-6 response of macrophages. Cells were treated with vehicle (Veh), IAld (0.25 mM), and/or CH-223191 (CH22) (10 μM), as indicated, 1 h before the addition of Mtb sonicate (10 μg/mL) followed by 16 h incubation. The expression of IL-1β (**a**) and IL-6 (**b**) mRNA relative to the untreated control (Fold Induction) is shown (*n* = 3). Data reported is derived from a representative experiment (Mean ± SEM) whose results are consistent with at least 3 independent experiments. Statistical difference was determined by an ordinary one-way ANOVA with post-hoc test for multiple comparisons. Two tailed *p*-value: NS = Not significant, ** *p* ≤ 0.01, *** *p* ≤ 0.001 and **** *p* ≤ 0.0001.

The potential role of AhR in mediating the observed inhibitory effect of IAld on the inflammatory response of Mɸ to Mtb was elucidated. RAW cells treated with CH-223191 (CH22), a known inhibitor of AhR commonly used to identify AhR-dependent mechanisms, along with Mtb, showed increased IL-1β expression (*p* = 0.0012) (Figure 2a) but unaltered IL-6 expression (Figure 2b). RAW cells treated concomitantly with IAld and Mtb exhibited reduced IL-1β and IL-6 expression compared to Mtb-treated controls. The addition of CH-223191 partially reversed IAld-mediated suppression of Mtb-induced IL-1β expression but did not reverse IAld-mediated suppression of IL-6 expression. Therefore, inhibition of AhR by CH-223191 caused only a partial reversal of IAld-mediated inhibition of IL-1β, whereas no such effect was observed for IL-6 expression. 

### 2.3. IAld-Mediated Inhibition of Pro-Inflammatory Cytokine Production by RAW Cells Was Not Associated with MyD88-Dependent Activation of Canonical NF-κB or MAPK Pathways

**Figure 3:**
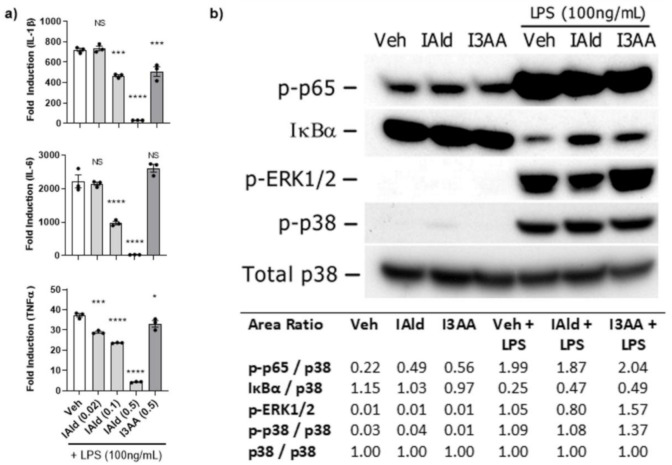
IAld-mediated Inhibition of pro-inflammatory cytokines apparently was not via MyD88-dependent signaling pathways. The effect of IAld and I3AA on the inflammatory response of RAW cells stimulated with lipopolysaccharide (LPS) (100 ng/mL) was assessed. (**a**) The transcription of pro-inflammatory cytokines was measured by qRT-PCR at 2 h after stimulation. Statistical difference was measured by ordinary ANOVA with post-hoc test for multiple comparisons. Statistical differences between the respective controls (Veh) and the treatment groups are indicated by the mark above each bar on the graph. Two tailed *p*-value is shown; NS = not significant, * *p* ≤ 0.05, *** *p* ≤ 0.001, **** *p* ≤ 0.0001. Data are from one experiment representative of three separate experiments (**b**) Activation of canonical inflammatory pathways was assessed by immunoblots from lysates collected after stimulation of cells with LPS (100 ng/mL) alone or with LPS in the presence of either IAld or I3AA (each at 0.5 mM). The lysates were collected from cells after 1 h of treatment with Veh, IAld or I3AA alone and from cells treated in parallel with an additional 15 min exposure to LPS. The densitometry ratio of each band after stimulation with medium only or LPS and the loading control (total p38) is reported. Blots are from a single representative experiment. IAld and I3AA exerted no consistent change in any of the tested signaling events between multiple independent experiments.

To obtain further mechanistic insights into the differential effect of IAld and I3AA on the pro-inflammatory cytokine response of RAW cells, we examined whether IAld and I3AA could influence canonical inflammatory pathways, namely NF-κB and MAPK. In these experiments, we utilized LPS, a TLR4 agonist, for activation of RAW cells because the signaling kinetics and integrated regulation of NF-κB and MAPK pathway are well defined for this model system. Notably, the addition of IAld inhibited the induction by LPS of not only IL-1β (0.5 mM; *p* < 0.0001) and IL-6 (0.5 mM; *p* = 0.0004), as was seen in Mtb-treated cells (Figure 1) but also of TNFα (0.5 mM, *p* = < 0.0001) compared to LPS-treated controls (Figure 3a). In contrast to the inhibitory effect of IAld, I3AA (0.5 mM) had no or little effect on LPS-induced expression of IL-6 or TNFα but it did reduce LPS- induced IL-1β (*p* = 0.0005), although to a lesser extent than IAld at the same concentration of 0.5 mM. 

With regard to LPS-induced MyD88-dependent signaling events, as expected, activation of the NF-κB pathway in LPS-treated cells was evident from the rapid increase in phosphorylation of p65 (Ser536) and parallel degradation of IκBα by 15 min (Figure 3b). In cells treated with LPS in the presence of IAld or I3AA (both at 0.5 mM), the phosphorylation of p65 was similar to that of LPS-treated controls. Additionally, in some experiments the level of IκBα measured in cells treated with LPS in the presence of IAld or I3AA showed slight differences compared to LPS-treated controls but no consistent difference was ascertained from multiple independent analyses. Importantly, this lack of difference in indicators of NF-κB activation after LPS was evident at 0.5 mM of IAld or I3AA. The same concentration that both IAld or I3AA showed inhibition in expression of pro-inflammatory cytokines (IL-1β, IL6 and TNFα) in response to LPS. Similarly, activation of MAPK pathways was evident from the phosphorylation of both ERK1/2 (Thr202/Thy204) and p38 (Thr180/Thy182) by 15 min after treatment of RAW cells with LPS. However, no difference in the level of phosphorylation of the MAPKs was observed in cells treated with LPS in the presence of IAld or I3AA compared to LPS-treated controls. Thus, neither IAld nor I3AA inhibits these three MyD88-dependent signaling pathways induced by LPS at concentrations at which they significantly inhibit LPS-induced expression of pro-inflammatory cytokines.

### 2.4. Modulation of RANKL-Induced Differentiation of RAW Cells into Osteoclasts by IAld But Not I3AA

**Figure 4:**
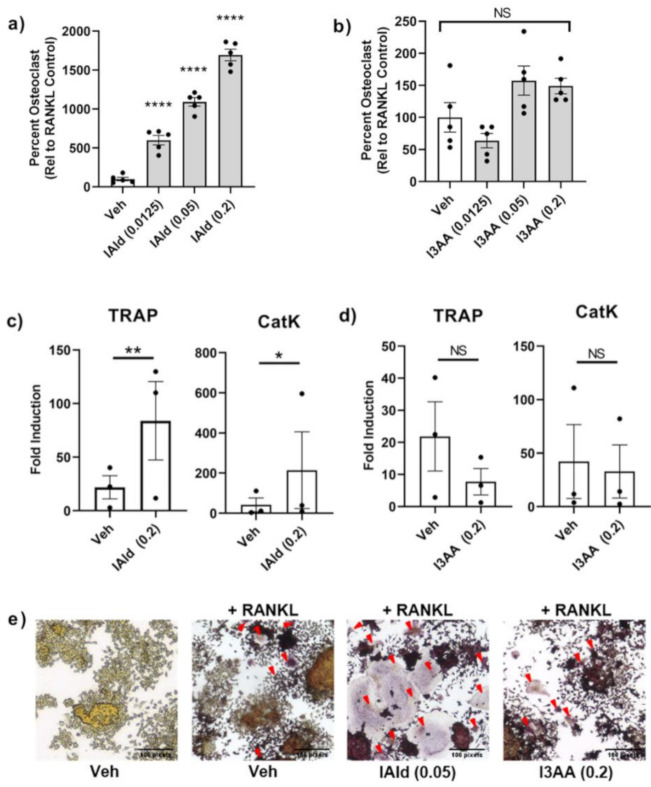
IAld enhanced the bone remodeling process of osteoclastogenesis. RAW cells were differentiated with receptor activator of NF-κB-ligand (RANKL) alone or with RANKL in the presence of either IAld or I3AA (0.0125–0.2 mM) as described in Methods. (**a**,**b**) Cells were fixed and then stained for tartrate-acid phosphates (TRAP) expression on d 5 and the number of osteoclast-like cells were enumerated from multiple regions of each well (*n* = 5). Data was reported as the percentage of osteoclast-like cells relative to the RANKL-treated controls (Mean ± SEM) from a representative experiment. Statistical difference relative to RANKL-treated control cells was determined by ordinary one-way ANOVA with post-hoc test for multiple comparisons. (**c**,**d**) The transcription of osteoclast genes TRAP and CatK on d 5 is reported from the combined results (Fold Induction) of 3 independent experiments (*n* = 3). Statistical difference was determined by paired Student’s *t* test. Indicators of statistical difference Two-tailed *p*-value: NS = Not significant, * *p* ≤ 0.05, ** *p* ≤ 0.01, **** *p* ≤ 0.0001. (**e**) Representative 20× magnification images taken from d 5 TRAP-stained cells: untreated, RANKL-treated control and cells treated with RANKL in the presence of IAld (0.05 mM) or I3AA (0.2 mM). Osteoclast-like cells are indicated by a red arrow. All results depict consistent trends observed in multiple independent experiments.

We tested further whether IAld or I3AA might directly regulate stromal remodeling of bone independent of their potential regulation of the upstream innate inflammatory response. The differentiation of osteoclast-like cells (cells that stain positive for TRAP and have 3 or more nuclei) from RAW cells treated with RANKL is a well-established model. Cells treated with IAld and RANKL (both added on d 0 and refreshed on d 3) had markedly increased numbers of osteoclast-like cells compared with RANKL-treated controls on d 5 by as much as ~16 times (0.2 mM, *p* < 0.0001) (Figure 4a). This pro-osteoclastogenic effect of IAld was observed repeatedly at a concentration as low as 0.0125 mM (*p* < 0.0001) with 2-4 times the number of osteoclast-like cells compared to RANKL-treated controls (Figure 4a and Figure S4) but no effect was observed at 0.003 mM of IAld (Figure S4). Additionally, cells treated concomitantly with IAld and RANKL showed increased expression of key enzymes involved in bone resorption activities, TRAP (*p* = 0.007) and CatK (*p* = 0.030), compared to RANKL-treated controls (Figure 4c). In contrast to the marked pro-osteoclastogenic effect of IAld, I3AA had no significant effect on either osteoclast-like cell formation (0.2 mM, *p* = 0.175) or on the expression of TRAP and CatK (Figure 4b,d). The pro-osteoclastogenic effect of IAld was further evident from both the increased number as well as overall surface area covered by osteoclast-like cells as shown in the representative images taken; whereas, the morphology of osteoclast-like cells treated concomitantly with I3AA and RANKL are similar to those formed with RANKL alone (Figure 4e).

### 2.5. Differential Regulation of Endothelial Cell Tube Formation by I3AA and IAld

Often in parallel with the remodeling of bone in the arthritic joint, there is excessive angiogenesis or blood vessel formation. We examined the impact of IAld and I3AA on in vitro endothelial cell (HUVEC) 2D-tube formation, a well-established model system for studying angiogenesis. VEGF-treated HUVEC seeded on Matrigel form distinct capillary-like tubes and complete looping structures by 8 h of treatment and these were stable even at 16 h. By 24 h after seeding of HUVEC, the integrity of structures is compromised even in VEGF-treated controls, as expected; therefore, quantification of tubes after 16 h of seeding cells was avoided. 

**Figure 5:**
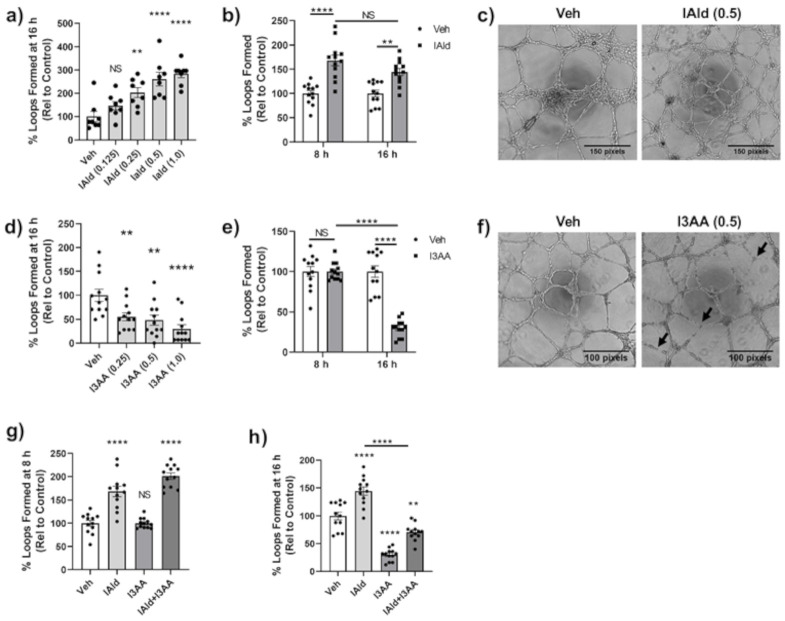
IAld and I3AA differentially influenced angiogenesis. human umbilical vein endothelial cells (HUVEC) were seeded on to Matrigel with VEGF (20 ng/mL) to perform a 2D Matrigel assay in which complete capillary-like structures (loops) were enumerated after seeding. Vehicle (Veh), I3AA, and/or IAld was added 1 h after seeding cells. Data was reported as the percent of loops formed relative to VEGF-treated controls. Combined results (Mean ± SEM) of data from different regions (*n* = 8–12) are shown, where data of each region is shown as a dot (**a**,**d**,**g**,**h**), or as a dot (Veh) or a square ((**b**), IAld; (**e**), I3AA). The dose effect (mM) on loop formation following treatment with IAld (**a**) or I3AA (**d**) at 16 h is shown. Also shown is the percentage of loops relative to VEGF-treated controls at both 8 h and 16 h with IAld (**b**) and I3AA (**e**) (both at 0.5 mM). Phase contrast 4× magnification images at 16 h after seeding of cells depicts loop formation in VEGF-treated control cells vs cells additionally treated with IAld (**c**) and I3AA (**f**) (both at 0.5 mM). The black arrows indicate tubes that have dissociated. The percentage of loops formed relative to VEGF-treated control cells when treated additionally with I3AA, IAld or both (both at 0.5 mM) at 8 h (**g**) and 16 h (**h**) are shown. Statistical differences were determined on unnormalized data by ordinary one-way ANOVA with post-hoc test for multiple comparisons (**a**,**d**,**g**,**h**) and by Two-way ANOVA with post-hoc test for multiple comparisons (**b**,**e**). NS = Not significant, * *p* ≤ 0.05, ** *p* ≤ 0.01, **** *p* ≤ 0.0001. Data are from one experiment representative of at least three separate experiments.

HUVEC treated concomitantly with IAld and VEGF formed significantly more loops at 16 h after seeding at a concentation as low as 0.25 mM of IAld (*p* = 0.007) compared to VEGF-treated control cells (Figure 5a). The percent increase in loops formed by concomitant treatment with IAld (0.5 mM) and VEGF at 8 h was equivalent to the percent increase at 16 h after seeding (*p* = 0.244) compared to VEGF-treated controls at the same times (Figure 5b). Therefore, the time of exposure to IAld did not appear to be a significant factor in its pro-angiogenic effect. The representative image depicts the relative increase in loop formation at 16 h compared to VEGF-treated control cells (Figure 5c). In contrast to the pro-angiogenic effect of IAld, I3AA exerted an anti-angiogenic effect (Figure 5d–f). This anti-angiogenic effect was dose dependent but a disruption of tube formation was still measured at the lowest concentration of I3AA tested (0.25 mM, *p* = 0.010) (Figure 5d). This effect of I3AA at 0.5 mM was highly time dependent whereby at 8 h after seeding, the percentage of loops formed in the presence of I3AA and VEGF was equivalent to that of controls but a profound inhibitory effect of I3AA on the stability of tubes was marked by their rapid dissociation at ~16 h after seeding (Figure 5e). This anti-angiogenic effect observed at 16 h is evident in the representative image shown, in which dissociated tubes are marked (Figure 5f). Even at 1 mM, I3AA was non-toxic to HUVECs as measured by the MTT assay (Figure S2) suggesting that induction of cell death was not the likely cause of the observed anti-angiogenic effect of I3AA. 

The temporal effect of I3AA on tube formation mentioned above was further evidenced when HUVEC were treated with a mixture of IAld and I3AA. The percent increase in loops relative to the VEGF-treated control cells at the early time point (8 h) was comparable in cells treated additionally with concomitant IAld and I3AA to those treated with IAld and no I3AA. Tube dissociation in cells treated concomitantly with IAld and I3AA at 16 h was similar to that seen with I3AA and no IAld (Figure 5g,h). 

### 2.6. AhR Antagonist Inhibited the Anti-Angiogenic Effect of I3AA But Not the Stomal Remodeling Effect of IAld on Endothelial Cells and Osteoclasts

**Figure 6:**
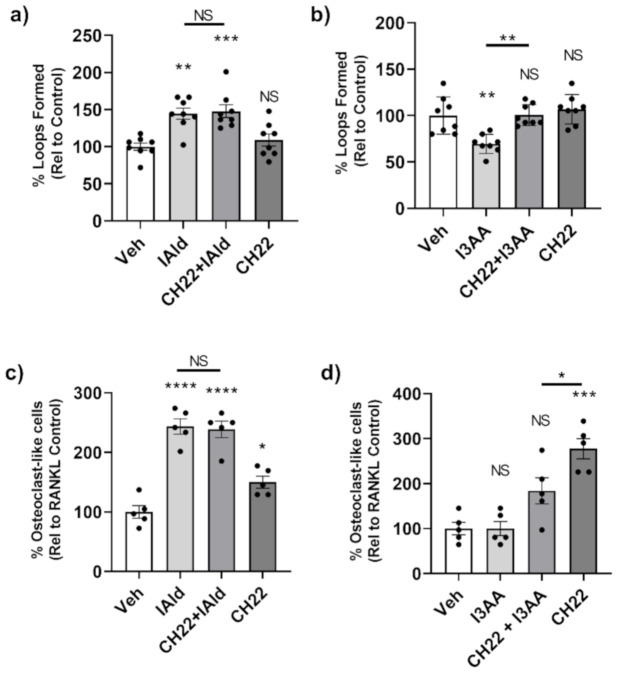
AhR antagonist CH-223191 inhibited the disruption of angiogenesis by I3AA but failed to alter the effect of IAld on angiogenesis and osteoclastogenesis. (**a**,**b**) HUVEC were seeded onto Matrigel with VEGF (25 ng/mL). After 1 h, cells were additionally treated with either IAld (0.5 mM) (**a**), I3AA (0.5 mM) (**b**), CH-223191 (3 μM) or a combination as indicated. The percentage of loops relative to VEGF-treated control cells (Veh) is reported (*n* = 8). (**c**,**d**) RAW cells were treated RANKL alone or RANKL in addition with IAld (0.05 mM) (**c**), I3AA (d), CH-223191 (10 μM) or a combination as indicated. The percentage of osteoclast-like cells relative to RANKL-treated control cells (Veh) was reported (*n* = 5). Statistical difference was determined on unnormalized data of either the number of loops formed (**a**,**b**) or from the number of osteoclast-like cells (**c**,**d**) by Ordinary one-way ANOVA with post-hoc test for multiple comparisons. Indicators of statistical different are between the respective controls (Veh) of the experimental type and the treatment group for which the mark is above, unless otherwise indicated by a bar. NS = not significant, * *p* ≤ 0.05, ** *p* ≤ 0.01, *** *p* ≤ 0.001, **** *p* ≤ 0.0001. Data are from one experiment representative of several separate experiments.

We again used CH-223191, an AhR antagonist, to assess the posssible involvement of AhR as a mediator of the effects of IAld and I3AA on the stromal remodeling events mentioned above. We first tested if CH-223191 alone had any effect on tube formation by HUVEC; no measurable effect was observed (Figure 6a,b). There was no measurable difference in tube formation from cells treated concomitantly with CH-223191 and IAld compared to those treated with IAld alone (*p* = 0.999) (Figure 6a). Therefore, CH-223191 did not alter the observed pro-angiogenic effect of IAld. However, when combined with I3AA, CH-223191 inhibited the disruptive effect of I3AA on tube formation (*p* = 0.001) (Figure 6b). At 16 h, HUVEC treated with both CH-223191 and I3AA had an equivalent number of loops as controls (*p* > 0.999), whereas cells treated with I3AA alone had about a third (*p* < 0.0002) the number of complete tubes compared to the control. Therefore, CH-223191 reversed I3AA-mediated tube dissociation. 

With regard to osteoclasts, the number of osteoclast-like cells formed in the presence of both CH-223191 and IAld was comparable to that formed in the presence of IAld alone (*p* = 0.999) (Figure 6c). This is despite the observation that CH-223191 alone increased the number of osteoclasts relative to controls (*p* = 0.046). Taken together, AhR antagonism did not display any measurable effect on the IAld-mediated osteoclastogenic effect. As mentioned above, I3AA alone did not have any effect on osteoclast generation; however, the number of osteoclast-like cells formed in the presence of I3AA and CH-223191 was significantly less than with CH-223191 alone (*p* = 0.0321) (Figure 6d), suggesting again that I3AA can modulate the AhR signaling pathway.

## 3. Discussion

Increasing evidence points to a role that the gut microbiota has in the maintenance of a healthy immune system. Although not definitive, there is also increasing evidence for the role of a ‘dysbiotic’ microbiome in contributing to the etiopathogenesis of rheumatic diseases [34,35,36,37], cancer [38] and overall health decline [19]. Immense progress has been made in characterizing the human microbiome, which was made possible by efforts of the Metagenomics of the Human Intestinal Tract consortium (Meta-HIT) and Human Microbiome Project (HMP) [39,40]. This has allowed for the identification of imbalances among different microbial species in the gut that are linked to diseases. Accordingly, probiotics aimed at re-setting the balance of species in the gut microbiota are being explored for health maintenance as well as therapeutic agents in autoimmunity, including RA [41,42,43,44,45] and other diseases. However, this effort has been fraught with difficulties for various reasons not limited to the highly dynamic nature of the gut-microbiome and fluctuating metabolic state that is highly influenced by individual factors. As such, alternative approaches (i.e., pre-biotics, post-biotics, synbiotics and microbiota-derived bioactive metabolites) are being investigated as alternative strategies for harnessing the beneficial aspects of a healthy microbiota to prevent disease and complement traditional autoimmune and cancer treatments. 

Evidence is rapidly growing to suggest that the bioactivities of microbiota-derived metabolites are a major mechanism by which microbiome is linked to host health and disease [16,46]. Of the microbiota-derived metabolites, those of the short chain fatty acid (SCFA) class have been rigorously characterized in a host of disease contexts including arthritis and metabolic syndrome, which is disproportionately high in rheumatic disease patients [47,48]. In contrast, this study is focused on the comparative analysis of two microbiota-derived metabolites of tryptophan within the class of indole-derivatives, IAld and I3AA and their potential to regulate innate inflammatory cytokines and stromal remodeling processes (osteoclastogenesis and angiogenesis) that contribute to joint pathology in RA. Indole-derivatives known to be produced by the microbiota have received some attention as being reduced in RA compared to OA synovial fluid [21] but their full implications for disease remain to defined. Our results highlight novel findings regarding the differential bioactivities of IAld *versus* I3AA against both the innate cytokine response and stromal remodeling, as well as the relative role of AhR versus non-AhR cell signaling pathways in these outcomes. 

Pro-inflammatory cytokines such as IL-1β, IL-6 and TNFα are the major mediators of arthritic inflammation in RA [1,2]. TLR2 and TLR4 responses were shown to regulate the cytokine milieu in human RA synovial explants [49]. In this regard, synovial-infiltrating Mɸ are a vital source of these cytokines in a diseased joint in RA. The TLRs expressed on the surface of Mɸ can be activated by various ligands including peptidoglycans, like those from Mtb and LPS (both Mtb and LPS are used in this study). We observed that IAld but not I3AA, reduced the expression of pro-inflammatory cytokines IL-1β and IL-6 by RAW cells (murine Mɸ) in response to stimulation by Mtb. Interestingly though, IAld significantly decreased the expression of TNFα by murine Mɸ, in addition to IL-1β and IL-6, in response to LPS. It is worth noting that TLR2 and TLR4 have been shown to play differential roles in the mycobacterium-induced response of Mɸ [32]. Unexpectedly, under identical conditions, the expression of VEGF was slightly increased in response to IAld, highlighting the potentially pleiotropic effects of IAld on processes downstream of the innate inflammatory response in RA. IAld was capable of inhibiting innate inflammatory cytokines in response to fibroblasts as well, which further points to its potential anti-inflammatory activity. These cytokines not only promote inflammation, they also drive mesenchymal processes as upstream mediators of osteoclastogenesis and angiogenesis. Therefore, novel approaches of targeting these cytokines, including administration of microbial-derived metabolites, may prove beneficial in arthritis therapy or as complementary approaches for disease management. 

Microbial indole-derivatives, including IAld, have been shown to ameliorate EAE and colitis in an AhR-dependent manner [23,24]. We observed that IAld reduced expression of proinflammatory cytokines IL-6 by murine Mɸ in response to stimulation by Mtb and that concomitant antagonism of the AhR with CH-223191 failed to suppress IAld-mediated suppression of IL-6. However, AhR antagonism directly enhanced IL-1β expression in response to Mtb treatment, in keeping with the finding that disrupting AhR in mouse peritoneal Mɸ leads to increased IL-1β expression during inflammation [50]. We speculate that the partial reversal by CH-223191 of IAld-mediated IL-1β suppression in reponse to Mtb was likely owing to increased IL-1β expression by CH-223191, not direct inhibition of the IAld-mediated effect. In an animal model of GVHD, the role of AhR in mediating the ameliorative effect of IAld was found inconclusive and evidence of the AhR response following IAld administration was not detected [51]. Additionally, studies directly analyzing IAld and I3AA in human colonocytes and human primary hepatocytes showed that these metabolite are potentially weak or ineffective AhR agonists [30,31], with I3AA showing greater agonistic effect in the human colonocytes. These findings suggest that IAld may have additional non-AhR dependent activities that in part facilitate its potential to regulate human health and disease. 

In human RA synovial tissue, which contains Mɸ expressing elevated TLR2 and TLR4 [5], TLR2 and TLR4 signaling contributed to the increased expression of inflammatory mediators and dominant negative MyD88 expression was shown to reduce TNFα, IL-6 and VEGF expression, without effecting IL-1β expression [49]. In our study, to assess any likely involvement of the NF-κB and MAPK pathways, which are downstream of TLR4-mediated MyD88 activation, in the observed inhibition of cytokine response, we used the prototypic TLR4 ligand, LPS, to activate RAW cells. However, no indication of regulation of these pathways by IAld or I3AA was observed. However, these findings do not rule out that the MyD88-response might be regulated downstream of these signaling events. For instance, the binding of MyD88-dependent transcription factors to promoter regions of the inflammatory genes or epigenetic changes may account for why certain cytokine and other genes are inhibited by IAld, while others were found upregulated. 

Stromal remodeling of bone and blood vessels plays a critical role in RA pathogenesis [1,4]. Indeed, resorption of bone in RA is the predominant factor in loss of mobility and chronic pain. With regard to osteoclastogenesis, our results showed that IAld promotes this process, whereas I3AA did not display any measurable effect. IAld enhanced osteoclast-like cell formation and related gene transcription of *CatK* and *TRAP* at concentrations as low as ~10–20 μM. To our knowledge, this would be first report describing a direct effect of IAld at a similar concentration. This observation contrasts with other reports where in vitro effects of IAld are described at 10 to 100 times higher concentration [24]. Additionally, AhR antagonism exerted a mild pro-osteoclastogenic effect. However, other similar studies on RAW cells have indicated that CH-223191 may have no effect or even a mild anti-osteoclastogenic effect [52,53]. Regardless, the pro-osteoclastogenic effect of IAld was not affected by AhR antagonism. The implications of the pro-osteoclastogenic effect of IAld at relatively lower concentrations deserve further attention in the context of RA and other diseases involving bone resorption. 

To our surprise, IAld and I3AA had opposing effects on endothelial tube formation, an indicator of angiogenic activity. IAld significantly upregulated tube formation, which in theory, could be detrimental within the synovial tissue if blood vessel invade the synovial lining and support the inflammatory response. In contrast to IAld, an anti-angiogenic effect of I3AA was observed. In addition, a time-dependent interplay between IAld and I3AA concomitant treatment was observed. These findings highlight the potentially differential outcomes in arthritic severity depending on the relative levels of these two indole-derivates by acting at different stages of blood vessel formation [54]. Additionally, we observed that CH-223191 failed to suppress the pro-angiogenic effect of IAld but the anti-angiogenic effect of I3AA was reversed by CH-223191. These findings lend further support for I3AA as an anti-angiogenic agent, as has been reported with I3AA-induced inhibition of VEGF signaling [55]. Recently, it was shown in mouse colonic cells that I3AA began to exert AhR agonistic properties at 0.25 μM [30]. A separate AhR agonist, Benzo [a]pyrene (BAP) was also recently shown to exert anti-angiogenic effects on HUVEC [56]. Taken together, these findings illustrate the differential activities of IAld and I3AA on stromal remodeling processes that are relevant not only for RA but other diseases as well.

In summary, our results demonstrate that despite their structural similarity, IAld and I3AA display functional differences. IAld has anti-inflammatory activity but pro-osteoclastogenic and pro-angiogenic effects which apparently may involve mechanisms not dependent on the AhR. On the contrary, I3AA displays anti-angiogenic activity only that was clearly AhR-dependent. Thus, for angiogenesis, these two indole derivatives show opposing effects. To fully comprehend the implications of these findings, it is imperative to consider that stromal remodeling not only contributes to the disease pathology in RA but it also is involved in physiological processes of bone remodeling and blood vessel formation in a healthy individual. For example, the end plate of a growing bone not only needs more blood supply, as provided by angiogenesis but also requires osteoclastic activity to properly sculpt the rapid bone formation by osteoblasts. To this can be added the anti-inflammatory effects of IAld to keep under control any low or moderate level of inflammation caused by any subclinical infections or injury and so forth. The same effect on cytokines would help protect against arthritic inflammation in a diseased individual. However, excessive availability of IAld may aggravate the angiogenic and osteoclastogenic components of arthritis pathology. Nevertheless, the anti-angiogenic effect of I3AA may help counter the pro-angiogenic effect of IAld in a disease situation. We suggest that the relative abundance of IAld and I3AA derived from the gut microbiota may determine the beneficial vs. harmful outcome of the effects of these indole derivatives. In this context, correcting the dysbiosis or metabolite deficiency/excess might help control the physiological/pathological outcomes.

## 4. Materials and Methods

### 4.1. Cell Culture

RAW 264.7 (RAW) cells (ATCC, Manassas, VA, USA) were cultured in Dulbecco’s Modified Eagles Media XL (DMEM XL) (Quality Biological, Gaithersburg, MD, USA). Human Umbilical Vein Endothelial Cells (HUVEC) (ATCC) were cultured in Endothelial Cell Growth Media 211–500 (Cell Applications, San Diego, CA, USA). All culture media was supplemented with 10% heat-inactivated fetal bovine serum (FBS), Penicillin (100 units/mL), Streptomycin (100 μg/mL) and L-Glutamine 2 mM (Gibco, Gaithersburg, MD, USA). 

### 4.2. Chemicals and Reagents

Indole-3-acetic acid (I3AA), Indole-3-aldhyde (IAld) and CH-223191 (CH22) were purchased from MilliporeSigma (Billerica, MA, USA). All compounds were dissolved in dimethyl sulfoxide (DMSO) (MilliporeSigma). The Leukocyte Acid Phosphatase Kit was purchased from MilliporeSigma. Heat-killed *M. tuberculosis* HR37a (Mtb) was obtained from Becton, Dickinson and Company (Sparks, MD) and was then used to prepare a sonicate supernatant as described [57]. Protein-free, phenol/water-extracted *Escherichia coli* LPS K235 was prepared as described previously [56]. Recombinant human vascular endothelial growth factor peptide (VEGF_165_) and murine soluble receptor activated NF-κB ligand (RANKL) was supplied by Peprotech (Rockhill, NJ, USA). Growth factor-reduced Matrigel was purchased from Corning (Corning, NY, USA). Primary antibodies (anti-IκBα (44D4), anti-phosphorylated ERK1/2 (197G2), anti-phosphorylated p38 (D3F9), anti-phospho-p65 (D3H1) and anti-total p38 (9212) were from Cell Signaling (Beverly, MA, USA).

### 4.3. MTT Assay for Cell Viability

Cells were seeded in a 96-well plate one day prior to treatment with IAld or I3AA. RAW cells and HUVEC were treated for 24 h. The 3-(4,5-dimethylthiazol-2-yl)-2,5-diphenyltetrazolium bromide (MTT) was then added in accordance with the protocol for MTT Assay for Cell Viability and Proliferation (Millipore Sigma). Absorbance was read at 595 nm after 4 h using a Bio-Rad iMark reader. The absorbance relative to the vehicle control was reported (Mean ± SEM) and correlates with relative cell viability [58].

### 4.4. Determining the Effect of Indole Derivatives on RAW Cells Treated with Heat-Killed M. Tuberculosis (Mtb) Sonicate or LPS 

(a) RAW cells (8.0 × 10^4^ /well) were seeded in 96-well plates one day prior to treatment. Cells were stimulated identically in 6-8 wells with Mtb (10 µg/mL) for 16 h or with LPS (20 ng/mL) for 2 h before collecting RNA from wells containing identically treated cells for qRT-PCR analysis. To assess the role of AhR in mediating the effect of IAld and I3AA on transcription, CH-223191 was added along with the vehicle (Veh), I3AA or IAld, as indicated. Veh, I3AA, IAld, CH-223191 were added 1 h prior before the addition of Mtb at the indicated concentrations. (b) RAW cells (1.5 × 10^6^ cells/well) were seeded in 12-well plates a day prior to treatment with LPS (100 ng/mL) in the presence or absence of Veh, IAld (0.5 mM) or I3AA (0.5 mM). Cell lysates were collected for subsequent Immunoblot analysis from cells not treated with LPS or from cells at the indicated time points after addition of LPS. 

### 4.5. Examining the Impact of Indole Derivatives on Osteoclast Differentiation

Osteoclast-like cells were generated from RAW cells and counted as described [59]. RAW cells (1.4 × 10^4^ cells/cm^2^) were seeded the day prior to treatment with RANKL (100 ng/mL) in addition to either Veh, I3AA or IAld. To assess the role of AhR in mediating the effect of IAld and I3AA on osteoclast-like cell formation, CH-223191 was added in addition to Veh, I3AA or IAld, as indicated. Media and treatments were refreshed 3 days later. On day 5, cells were washed, fixed and stained for 1–2 h at 37 °C to detect tartrate-acid phosphates (TRAP) expression according to the manufacturer’s instructions. Brightfield images were taken using a Keyence Microscope (Osaka, Japan). The number of osteoclast-like cells (i.e., tartrate-resistant acid phosphatase-positive (TRAP) multinucleated (3+ nuclei) cells) were enumerated using ImageJ software (NIH). In parallel to staining, expression of osteoclast genes (i.e., TRAP and CatK) was also assessed in the treated cells by qRT-PCR.

### 4.6. HUVEC Tube Formation Assay and Regulation of Angiogenesis by Indole Derivatives

The tube formation assay was performed according to a previously reported procedure, with minimal modifications [60]. HUVEC were serum-starved overnight in culture with Endothelial Cell Basal Media (ECBM) 210–500 (Cell Applications, San Diego, CA, USA) containing 1% FBS before seeding at 2.5 × 10^4^ cells/well in with VEGF_165_ (25 ng/mL) in a 96-well plate (Costar) containing 30 µL of Matrigel. Wells were checked 30 min after seeding to assure equal seeding density. One hour after seeding, Veh, I3AA (0.25–1.0 mM) or IAld (0.125–1.0 mM) was added to the cells. To assess the role of the AhR pathway in mediating the effect of I3AA and IAld on angiogenesis, CH-223191 (3 µM) was added at the time of cell seeding along with VEGF. Phase contrast images from 4 regions of each well were taken 8 or 16 h later using a Keyence microscope (Osaka, Japan) and analyzed using ImageJ Software (NIH).

### 4.7. Quantitative Real-Time Polymerase Chain Reaction (qRT-PCR)

Total mRNA was isolated from cells using TRIzol (Invitrogen, Carlsbad, CA, USA), according to the manufacturer’s instructions. cDNA was prepared using the qScript cDNA Synthesis kit (Quantabio, Beverly, MA, USA). qRT-PCR was performed using Power SYBR Green PCR Master Mix (Applied Biosystems, Forster City, CA, USA). Primers were either designed in-house using the Primer Express 2.0 Program (Applied Biosystems) and based on those from the Harvard primer bank or obtained from a commercial source (Integrated DNA Technologies (IDT) and Millipore Sigma) (Table S1). mRNA expression values were normalized to the housekeeping gene hypoxanthine-guanine phosphoribosyl transferase (HPRT) and expressed as fold-change in expression relative to the untreated controls (fold-induction) using the 2^−ΔΔCT^ method [61].

### 4.8. Analysis of Cell Signaling by Immunoblot Analysis

Cells lysates were processed and immunoblots run similar to the described method [62]. Cells were lysed in a buffer (1% Triton ×100, 25 mM HEPES, 100 mM NaCl, 0.1% SDS) and placed on ice before freezing at −80 °C until further use. Cell lysates were separated by electrophoresis on a mini protean TGX gel (Biorad, Hercules, CA, USA) for subsequent transfer to PVDF membrane. The blots were then blocked with TBST (0.3% Tween) and 5% non-fat dry milk, washed three times with TBST (0.3% Tween) while shaking over the course of 1 h and then incubated overnight with the primary antibodies at 4 °C in either TBST 5% non-fat dry milk or 5% BSA. The following day, membranes were washed three times with TBST and then incubated with the anti-rabbit HRP-conjugated secondary antibody (Jackson Immunochemicals, West Grove, PA, USA) in TBST and 5% milk. Blots were developed following incubation with ECL Plus Western Blotting Detection Reagent (Amersham Bioscience, Piscataway, NJ, USA). Blots were stripped with Biorad western stripping reagent before subsequent reblotting. Total MAPK p38 protein was used as a loading control for normalization of densitometry analysis using ImageJ. The ratio of signaling intensity is indicated relative to p38.

### 4.9. Statistical Analysis

Statistical analysis of data was performed using GraphPad Prism (v8.4.3) software. Ordinary one-way analysis of variance (ANOVA) with the subsequent recommended Sidak or Dunnett’s post-hoc test for multiple comparisons of more than two groups when comparing one parameter. Two-way ANOVA with Sidak poc-hoc test for the additional comparison of time as a factor in the HUVEC tube formation assay. The paired or standard Student’s *t* test (two-tailed) was used to compare the results from two groups for data collected from multiple independent experiments or replicates of a single representative experiment, respectively. A P value of less than 0.05 was considered statistically significant. Data from a single experiment is representative and consistent with the results that were observed in multiple independent experiments.

## Data Availability

Data is contained within the article or supplementary material.

## Supplementary Materials

The following are available online at https://www.mdpi.com/1422-0067/22/4/2017/s1, Figures S1–S4; Table S1.
