# Supplementary material for: Microbiota-Derived Metabolites, Indole-3-aldehyde and Indole-3-acetic Acid, Differentially Modulate Innate Cytokines and Stromal Remodeling Processes Associated with Autoimmune Arthritis
Source: Int J Mol Sci. 2021 Feb 18;22(4):2017. doi: 10.3390/ijms22042017 (PMC7922345; doi:10.3390/ijms22042017)
Supplement: Supplementary file 1 [file ijms-22-02017-s001.pdf]

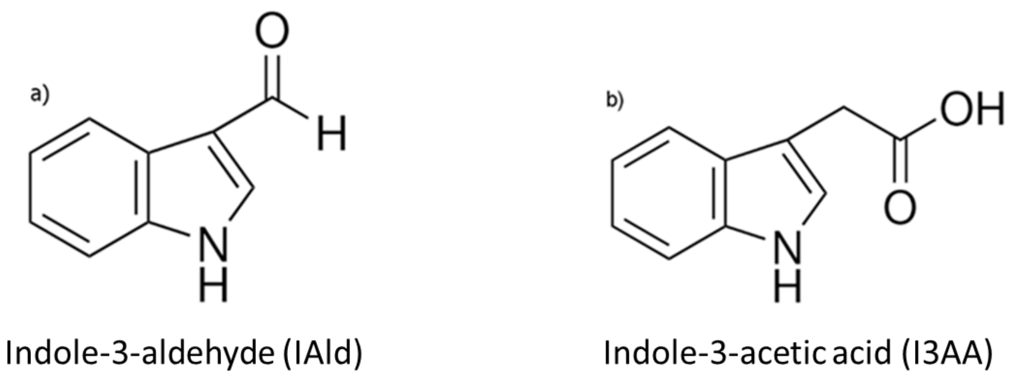

Figure S1. Structures of IAld and I3AA. The structures of IAld (a) and I3AA (b) are shown.

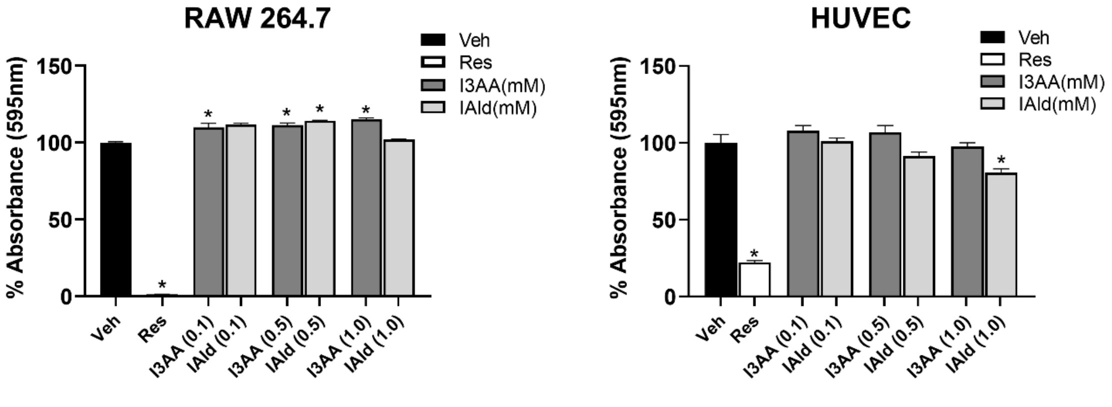

Figure S2. Assessment of cellular cytotoxicity of I3AA and IAld by MTT assay. HUVEC (a) or RAW (b) cells were treated for 24 h with either vehicle (Veh), indole-3-acetic acid (I3AA), or indole-3-aldehyde (IAld) at the indicated concentration (mM) followed by the addition of MTT. Results of a representative experiment (n = 3-4) are reported as percent absorbance (595 nM) (Mean ±SEM) relative to the vehicle (Veh) control. Resveratrol (Res) was used as a positive control for the cytotoxic effect. Even at 1mM, both IAld and I3AA showed minimal effects. Statistical difference was tested by ordinary one-way ANOVA with post-hoc test for multiple comparisons. Asterisk (*) indicates the conditions with $p < 0.05$.

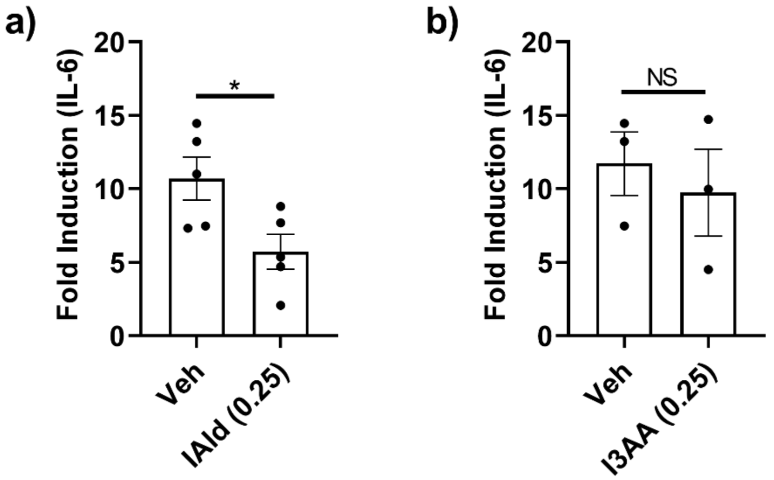

Figure S3. Indole-3-aldehyde (IAld) inhibits the production of IL-6 by NIH 3T3 cells in response to IL-1β. NIH 3T3 cells were treated with vehicle (Veh) or IAld (0.25 mM) (**a**) or with Veh or I3AA (0.25 mM) (**b**), 1 h before the addition of IL-1β sonicate (20 ng/mL), followed by a 24 h incubation. Gene expression relative to the untreated vehicle control (Fold Induction) is reported. Data reported are from the combined results (Mean ± SEM) of 4 independent experiments ($n = 4$) whose values are shown as individual dots. Statistical difference between two groups in each panel was determined by a paired Student's t test. Two- tailed p-value: NS = Not significant, * $p \leq 0.05$.

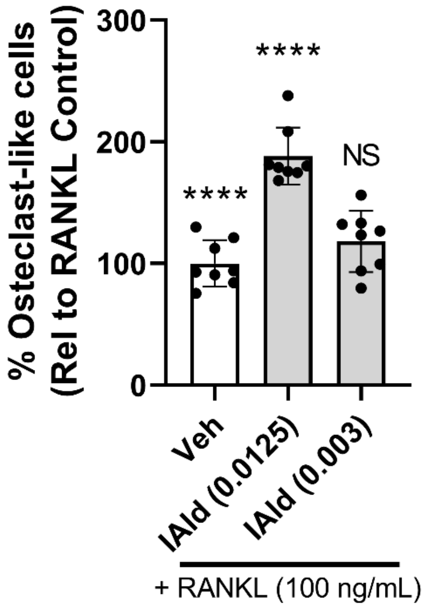

Figure S4. IAld enhanced the differentiation of RAW cells into osteoclasts. RAW cells were differentiated with receptor activator of NF-κB-ligand (RANKL) alone or with RANKL in the presence of either IAld or I3AA at the indicated concentration (mM) as described under Methods. Cells were stained for TRAP expression on d 5 and the number of osteoclasts were enumerated from 8 images taken of each well (n = 8). Combined results (Mean ± SEM) of data from different regions (n= 8) are shown, where data of each region is shown as a dot. Statistical difference relative to the RANKL control was determined by ordinary one-way ANOVA with Dunnett's correction for multiple comparisons. Not significant = NS, **** $p \leq 0.0001$

Table S1. Sequences of in-house designed primers.

Target	Source	Sequence Fwd	Sequence Rev
HPRT	Sigma	5' – TTGCTGACCTGCTGGATTAC – 3'	5' – ACTTTTATGTCCCCCGTTGACTGAT – 3'
IL-1β	IDT	5' – GGTCAAAGGTTTGGAAGCAG – 3'	5' – TGTGAAATGCCACCTTTTGA – 3'
IL-6	Sigma	5' – GATGGATGCTACCAAACTGGA – 3'	5' – TCTGAAGGACTCTGGCTTTG – 3'
TNFα	Sigma	5' – GACCCTCACACTCAGATCATCTTC – 3'	5' – CCACTTGGTGGTTTGCTACGA – 3'
COX-2	IDT	5' – TGCCTGGTCTGATGATGTATG – 3'	5' – GGGGTGCCAGTGATAGAGTG – 3'
CatK	Sigma	5' – CTTCCAATACGTGCAGCAGA – 3'	5' – GCCGTGGCGTTATACATACA – 3'
TRAP	IDT	5' – ACGGCTACTTGCGGTTTCACTA – 3'	5' – GTGTGGGCATACTTCTTTCCTGT – 3'
